# Expansion of Anti-Mesothelin Specific CD4^+^ and CD8^+^ T Cell Responses in Patients with Pancreatic Carcinoma

**DOI:** 10.1371/journal.pone.0088133

**Published:** 2014-02-10

**Authors:** Yuan Chen, Lakshmana Ayaru, Sanju Mathew, Emma Morris, Stephen P. Pereira, Shahriar Behboudi

**Affiliations:** 1 The Institute for Liver and Digestive Health, University College London, London, United Kingdom; 2 Department of Medicine, Imperial College London, London, United Kingdom; 3 Institute of Immunity and Transplantation, University College London and the Royal Free London NHS Foundation Trust, London, United Kingdom; University of Szeged, Hungary

## Abstract

We aimed to assess the status of naturally occurring CD4^+^ and CD8^+^ T cell responses to a tumour associated antigen, Mesothelin, in patients with pancreatic carcinoma and study the effects of elevated IL-10 on Mesothelin-specific T cell responses. For that sake, short term T cell lines were generated from PBMCs of 16 healthy controls, 15 patients with benign pancreatic diseases and 25 patients with pancreatic carcinoma and Mesothelin-specific CD4^+^ and CD8^+^ T cell responses were analysed using intracellular cytokine assays for IFN-γ. Plasma levels of IL-10 and Mesothelin were measured using cytometric bead array and ELISA assay, respectively. The blocking assays were performed to assess the effects of IL-10 on Mesothelin-specific T cell responses. Here, we demonstrate that the plasma levels of Mesothelin and IL-10 are significantly increased in patients with pancreatic carcinoma. Additionally, we found that (a) Mesothelin-specific T cell responses are significantly expanded in cancer patients (p = 0.0053), (b) the multifunctional CD4^+^ T cell response is directed toward a broad repertoire of epitopes within the Mesothelin protein. (c) Mesothelin-specific CD4+ T cell response is directly inhibited by elevated IL-10 in cancer patients. These data provides evidence for the use of Mesothelin as an immunogen for tumour-specific T cell response.

## Introduction

Pancreatic cancer (PC) is a significant cause of tumour-related death worldwide, which ranks the 10^th^ commonest cancer in men and the 11^th^ in women [1], and stands as the 4^th^ leading cause of cancer-linked mortality in both the USA [2] and Europe [3]. Important risk factors for this disease include age, smoking, obesity, family history chronic pancreatitis and diabetes [4]–[9]. Early diagnosis may increase the chance of curative resection of pancreatic cancer [10]. Surgical resection such as pancreaticoduodenectomy (Whipple technique) is suitable for patients with localized pancreatic cancers [11], but is only appropriate for up to 15–20% of patients [12]. Nearly 30% of PC patients have locally advanced disease by the time of diagnosis, with a further 50% patients carrying metastatic deposits [13]. Gemcitabine or folfirinox-based regimens are the treatment of choice for unresectable or metastatic pancreatic cancer and are also used in the neoadjuvant/adjuvant setting [14]–[16], yet, over the last 30 years there has been little improvement in survival of PC patients with an overall 5 year survival 2–6% [2]. Thus, innovative diagnostic and therapeutic strategies are urgently required.

Cell-based antitumor immunotherapy is a promising strategy for the treatment of some cancers and it is also being considered for the treatment of pancreatic cancer. Activated CD8^+^ T cells have been shown to migrate to the tumour microenvironment and cause lysis of tumour cells mediated by granzyme B, perforin or Fas[17]. Effector Th1 type helper CD4^+^ T cells with the ability to secrete IFN-γ and TNF-α can act as regulators in anti-tumour immunity and provide ‘help’ for the development of potent anti-tumour CTLs [18], [19] by supporting their expansion and memory development. Activated tumour-infiltrating lymphocytes are observed in many types of tumours and relate to better prognosis [20], [21]. Successful antitumor T cells responses rely on recognition of tumour-associated antigens (TAAs) by T cells [22].

Mesothelin (MSLN) is a 40k-Da GPI-anchored glycoprotein expressed on the cell surface, which can be released by Phospholipase C [23]. In normal cells, MSLN expression is limited to the surface of a single layer of mesothelial cells located in pleura and peritoneum [24]. MSLN is overexpressed in many types of cancers, including ovarian, pancreatic/biliary, lung cancers and mesotheliomas [25]. Using a monoclonal antibody (5B2) directed against MSLN, expression was identified in 91–100% of pancreatic cancer tissue [26]–[28]. In addition, some studies also reported elevated levels of soluble MSLN in mesothelioma, ovarian cancer and pancreatic cancer [29]–[31]. The biological function of MSLN has not been clearly understood, but a MSLN knock-out mouse model showed no clear phenotypic or functional defects during development or with reproduction ability [32]. However, in mouse models of pancreatic cancer, MSLN overexpression was shown to promote metastasis and proliferation of tumour cells [33]. In animal models, the induction of anti-Mesothelin CD8+ T cell responses resulted in the reduction in tumour volume and prolonged survival in an orthotopic PC mouse model [33], [34], suggesting that MSLN is a tumour rejection antigen for pancreatic carcinoma. A pilot study further illustrated that an allogeneic GM-CSF secreting pancreatic cancer cells vaccine could stimulate MSLN specific T cells and that was favourable in gemcitabine-resistant advanced pancreatic cancer patients. Furthermore, this study demonstrated that MSLN specific CD8+ T cell response could be induced via cross presentation of tumour antigens in an immunotherapy approach that recruits antigen presenting cells to the site of vaccination [35]. Thus, MSLN is a possible biomarker for pancreatic cancer and target for T-cell based immunotherapy. There is very little information on naturally occurring MSLN-specific T cell responses in cancer patients. This study aimed to identify and functionally characterise MSLN-specific T cell responses and establish whether the elevation of immune-regulatory cytokines such as IL-10 can modulate this response.

## Materials and Methods

### Patients

The study was approved by the Central London REC 3 Research Ethics Committee, and all patients gave written, informed consent. The trial was run in accordance with the Declaration of Helsinki. Blood samples were obtained from patients at University College Hospital, Royal Free Hospital (UCL, London, UK), and Charing Cross Hospital (IC, London, UK). Mononuclear cells were isolated from peripheral blood samples of patients with pancreatic cancer (Table 1), benign pancreatic disease (Table 2) and age- and sex-matched healthy donors. Pancreatic carcinoma (PC) diagnosis was confirmed by standard cytopathology or histopathology after biopsy. The clinical staging of patients with pancreatic carcinoma was determined using the WHO histological classification of tumours of the exocrine pancreas [36].

**Table 1:** Pancreatic cancer patients' information.

ID of PC patients	Age (year)	Gender	Histological diagnosis	Grade	Plasma mesothelin (ng/ml)
PC01	63	F	adenocacinoma	IVB	21.8
PC02	77	M	Adenocacinoma with squamous differenciation	IVB	21.6
PC03	47	M	PDAC	III	74.0
PC04	80	M	Epithelial neoplasia	n.a.	53.4
PC05	66	F	PDAC	n.a.	13.8
PC06	80	F	PDAC	n.a.	20.1
PC07	72	M	adenocacinoma	n.a.	13.4
PC08	54	F	Poorly differentiated ademocarcinoma	III	n.a.
PC09	41	M	adenocacinoma	n.a.	13.3
PC10	71	F	morderately differentiated adenocarcinoma	II	34.9
PC11	86	F	adenocacinoma	IVA	
PC12	83	F	adenocacinoma	II	27.3
PC13	n.a.	F	moderatedly differentiated adenocarcinoma	n.a.	42.3
PC14	60	F	adenocacinoma	III	26.4
PC15	80	F	morderately differentiated adenocarcinoma	III	52.3
PC16	74	M	PDCA (not tolerate to chem.)	IVB	75.78
PC17	78	M	PDCA (in chem.)	IVB	27.034
PC18	67	F	postoperative ademocacinoma	Disease-free	18.17
PC19	94	F	adenocacinoma	IVB	40.1
PC20	76	M	PDAC	IVA	38.9
PC21	69	M	adenocacinoma	IVB	27.6
PC22	65	M	Recurrent adenocacinoma	IVA	100.956
PC23	61	M	Recurrent adenocacinoma	IVA	30.7
PC24	41	M	poor differentiation p ampilay cancer	IV	18.2
PC25	69	F	adenocacinoma	III	32.7

**Table 2:** Patients Demographic Information (Benign disease patients).

ID of control patients	Age (year)	Gender	Diagnosis	Plasma Mesothelin (ng/ml)
CON01	40	M	Chronic pancreatitis	56.7
CON02	50	F	Acute pancreatitis	31.6
CON03	77	F	mucinous cystic lesion	11.5
CON04	54	F	benign lesion in pancreas	17.1
CON05	32	F	recurrent pancreatitis	N.A.
CON06	50	F	Acute pancreatitis secondary to biliary duct stone	N.A.
CON07	63	M	Cystic disease	33.9
CON08	41	M	Chronic pancreatitis	62.5
CON09	82	M	Cystic disease	18.2
CON10	n.a.	F	Chronic pancreatitis	7.6
CON11	46	F	Chronic pancreatitis	11.3
CON12	24	M	Cystic disease, acute pancreatitis	0
CON13	59	F	Recurrent pancreatitis	31.0
CON14	50	M	Necrotic pancreatitis	19.1
CON15	53	F	Cystic disease	N.A.

### Mesothelin ELISA

Plasma Mesothelin (MSLN) concentration was measured by Quantikine (R&D Systems Europe, Ltd, Abingdon, UK) - a validated double determinant sandwich ELISA. Briefly, 5 µl plasma (1 in 10 diluted in assay diluent) was incubated in MSLN antibody pre-coated microplates at room temperature for 2 hours. After washing unbound elements, a MSLN-specific enzyme-linked monoclonal antibody was added and incubated for a further 2 hours, prior to adding substrate for 30 minute incubation. A microplate reader was used to determine optical density after stopping the development reaction.

### Cytometric bead array (CBA)

Concentrations of IL-10 in plasma samples were determined using the BD cytometric bead array human inflammatory cytokines kit (BD Biosciences, CA, US). In brief, beads coated with antibodies binding the above-mentioned cytokines were incubated with plasma for 3 hours in order to ‘capture’ cytokines. The samples were analysed by FACSCanto II flow cytometer (BD Biosciences) in the FL-3 channel using FCAP Array Software v3.0 (BD Biosciences).

### Peptide library

Peptides corresponding to the sequence of MSLN were synthesized by Mimotopes Pty Ltd. (Clayton Victoria, Australia). Fifteen amino acid long peptides, overlapping by five amino acids, were pooled into 7 peptide pools. Details of amino acid sequences are given in Table 3.

**Table 3:** Mesothelin-derived peptides.

Amino acid start	pool	sequence	Amino acid start	pool	sequence
1	1	MALPTARPLLGSCGT	321	4	WELEACVDAALLATQ
11	1	GSCGTPALGSLLFLL	331	4	LLATQMDRVNAIPFT
21	1	LLFLLFSLGWVQPSR	341	4	AIPFTYEQLDVLKHK
31	1	VQPSRTLAGETGQEA	351	4	VLKHKLDELYPQGYP
41	1	TGQEAAPLDGVLANP	361	4	PQGYPESVIQHLGYL
51	1	VLANPPNISSLSPRQ	371	4	HLGYLFLKMSPEDIR
61	1	LSPRQLLGFPCAEVS	381	4	PEDIRKWNVTSLETL
71	1	CAEVSGLSTERVREL	391	4	SLETLKALLEVNKGH
81	1	RVRELAVALAQKNVK	401	5	VNKGHEMSPQAPRRP
91	1	QKNVKLSTEQLRCLA	411	5	APRRPLPQVATLIDR
101	2	LRCLAHRLSEPPEDL	421	5	TLIDRFVKGRGQLDK
111	2	PPEDLDALPLDLLLF	431	5	GQLDKDTLDTLTAFY
121	2	DLLLFLNPDAFSGPQ	441	5	LTAFYPGYLCSLSPE
131	2	FSGPQACTRFFSRIT	451	5	SLSPEELSSVPPSSI
141	2	FSRITKANVDLLPRG	461	5	PPSSIWAVRPQDLDT
151	2	LLPRGAPERQ RLLPA	471	5	QDLDTCDPRQLDVLY
161	2	RLLPAALACWGVRGS	481	5	LDVLYPKARLAFQNM
171	2	GVRGSLLSEADVRAL	491	5	AFQNMNGSEYFVKIQ
181	2	DVRALGGLACDLPGR	501	6	FVKIQSFLGGAPTED
191	2	DLPGRFVAESAEVLL	511	6	APTEDLKALSQQNVS
201	3	AEVLLPRLVSCPGPL	521	6	QQNVSMDLATFMKLR
211	3	CPGPLDQDQQEAARA	531	6	FMKLRTDAVLPLTVA
221	3	EAARAALQGGGPPYG	541	6	PLTVAEVQKLLGPHV
231	3	GPPYGPPSTWSVSTM	551	6	LGPHVEGLKAEERHR
241	3	SVSTMDALRGLLPVL	561	6	EERHRPVRDWILRQR
251	3	LLPVLGQPIIRSIPQ	571	7	ILRQRQDDLDTLGLG
261	3	RSIPQGIVAAWRQRS	581	7	TLGLGLQGGIPNGYL
271	3	WRQRSSRDPSWRQPE	591	7	PNGYLVLDLSMQEAL
281	3	WRQPERTILRPRFRR	601	7	MQEALSGTPCLLGPG
291	3	PRFRREVEKTACPSG	611	7	LLGPGPVLTVLALLL
301	4	ACPSGKKAREIDESL	616	7	PVLTVLALLLASTLA
311	4	IDESLIFYKKWELEA			

### T cell culture and intracellular cytokine staining assay

PBMCs were re-suspended at a concentration of 1.5×10^6^/mL in RPMI 1640, 10% heat-inactivated FCS (Life technologies, Grand Island, NY), and 1% penicillin plus streptomycin (Sigma-Aldrich, St. Louis, MO). PBMCs were stimulated with peptide pools at a final concentration of 2 µmol/ mL for each individual peptide or irrelevant peptides, as negative control. We have selected the peptide concentration used in this study based on our previous T epitope mapping studies and reports from other researchers in the field. Recombinant IL-2 (25 UL/mL) was added on the 1^st^ day of culture and the cells were analysed after a total of 9-10 days culture. T-cell lines were re-stimulated with the same peptide pools for a further 5 hours in the presence of Brefeldin A. Cells were surface stained with anti-CD4 and anti-CD8 antibodies (BD PharMingen, Cowley, United Kingdom), then permeabilized and fixed using formaldehyde and saponin. After fixing, the cells were stained for intracellular cytokines with FITC-conjugated anti IFN-γ, FITC-conjugated anti-IL-2, and PE-conjugated anti-TNFα (R&D Systems, Abingdon, United Kingdom).

### Individual peptide simulation experiments

In some patients where MSLN-specific CD4^+^ T cell responses were observed and surplus PBMC were available for further testing, we performed additional experiments to identify the cognate peptides. PBMCs were cultured with responding peptide pool and rIL-2 in RPMI (as before) for 9-10 days. On day 9 or 10, these cells were further cultured for 5 hours in the presence of individual peptides from the relevant peptide pool, or with irrelevant peptide as control. The T cells were then stained with anti-CD4, anti-CD8 and IFN-γ prior to analysis by flow cytometry (as before).

### IL-10 blockade

We performed IL-10 blocking assays during peptide stimulation of PBMC from some patients that we had obtained larger number of cells. MSLN-specific T cell lines were generated in the presence of blocking antibodies to anti-IL10 (5 µg/mL) (eBioscience) and anti-IL-10R (10 µg/mL). On day 4, 150 µl supernatant was removed, and replenished with 150 µl of fresh medium with additional anti-IL-10 antibody 5 µg/ml, IL-10Ra (10 µg/ml), as well as IL-2 (25 UL/mL). The re-stimulation and intracellular cytokine staining at day 9–10 was performed as described above.

### Statistical analysis

Statistical analysis was performed using SPSS for windows 17.0. The Mann-Whitney U test (for 2 groups) and Kruskal-Wallis test (for 3 groups) analyses were applied to compare the plasma levels of MSLN and the 6 cytokines in pancreatic cancer patients compared to control subjects. The Chi-squared test was used to determine statistical significance of MSLN-specific CD4^+^ and CD8^+^ T cell response rates in cancer and control groups. The Spearman rank coefficient was utilized to check the correlation between plasma MSLN levels and T cell response levels. Kruskal-Wallis test was employed to analyse the levels of T cell response between PC, benign pancreatic disease patients and healthy volunteers. A significant interval was defined at 95%, or two tailed p-value ≤ 0.05.

## Results

### Plasma MSLN levels are increased in pancreatic cancer patients

**Figure 1:**
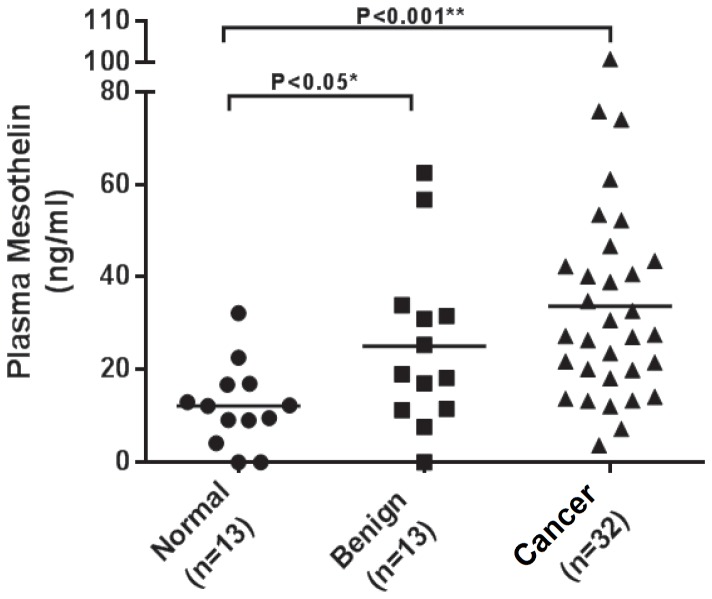
Plasma Mesothelin concentrations in different groups of patients. This figure shows plasma Mesothelin concentrations in pancreatic cancer patients (n = 32), patients with benign pancreatic disease (n = 13), or healthy controls (n  =  13) detected using an ELISA assay for human Mesothelin. The horizontal line displays the median value for each group. Mann-Whitney U test was used to compare the levels of plasma Mesothelin in different groups of patients.

A sandwich enzyme immunoassay using monoclonal antibody directed against MSLN was used to detect circulating MSLN levels. The method is described in detail in the Material and Methods section. Of a total 13 healthy controls, 11 (85%) had MSLN levels less than 20 ng/ml, with the median value at 12.0 ng/ml (ranged from 0 ng/ml to 32.2 ng/ml). Compared to healthy controls, the plasma MSLN levels were significantly elevated in patients with benign pancreatic disease (n = 13, p<0.05) (Figure 1), where the median concentration was 19.1 ng/ml (range: 0 to 62.5 ng/ml). The median plasma MSLN concentration in 32 pancreatic cancer patients was 27.4 ng/ml, with the minimum of 3.7 ng/ml and maximum of 101 ng/ml. Additionally, statistically significant differences were observed between soluble MSLN levels of PC patients and that of healthy volunteers (p<0.001), but no significant differences between the plasma MSLN concentration in the cancer patients and that of the benign pancreatic disease patients (p = 0.17) (Figure 1). Subset analysis between metastatic pancreatic cancer (stage IVB) compared to patients with benign pancreatic disease demonstrated no significant difference in soluble MSLN concentration between these groups (data not shown). These results suggest that circulating MSLN levels are significantly increased in patients with benign and malignant pancreatic disease.

### Plasma IL-10 levels are increased in pancreatic cancer patients

**Figure 2:**
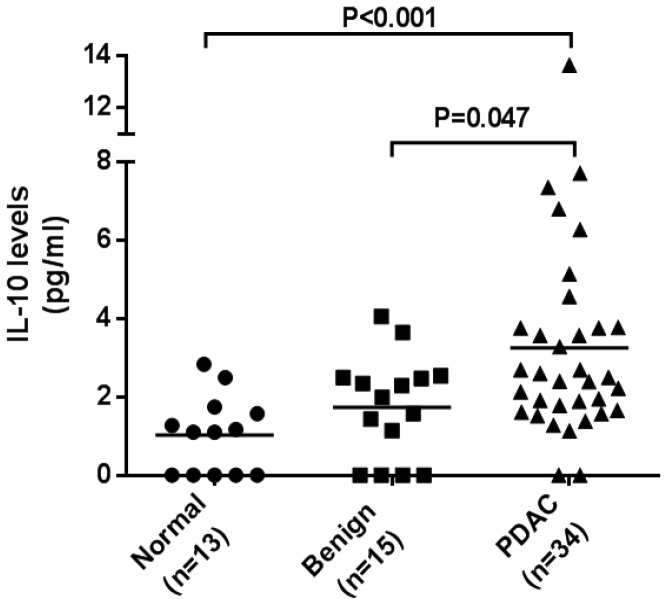
Plasma IL-10 concentration. This figure displays the plasma IL-10 levels observed in the different disease groups: pancreatic cancer patients (n = 34), patients with benign pancreatic disease (n = 15), or healthy controls (n  =  13). Median values of each group are presented by the horizontal lines. The plasma IL-10 levels was elevated in pancreatic cancer patients, with a median at 2.45 pg/mL (n = 34), which was significantly higher than both healthy donors (P<0.001), whose median was 1.10 pg/mL (n = 13), and benign pancreatic disease patients (P = 0.047), with median plasma IL-10 concentration at 1.99 pg/mL, (n = 15). There is no significant difference between normal volunteers and patients with benign pancreatic disease in the regards of plasma IL-10 levels (P = 0.14).

A cytometric bead array (CBA) assay was used to determine the concentration of IL-10 in plasma samples (as described in material and method section). IL-10 concentration was determined by flow cytometry after the generation of a standard curve derived from serial dilutions. Plasma IL-10 concentrations were significantly higher in patients with benign pancreatic disease compared to normal controls (p<0.001). Figure 2 shows the plasma IL-10 levels observed in the different disease groups (normal, benign disease and PC). Statistical comparisons were made using Mann-Whitney U test to determine the differences within the 3 subject groups. As we can see, plasma IL-10 concentrations was significantly increased in pancreatic cancer patients (median = 2.45 pg/mL, n = 34), with comparison to both benign pancreatic disease patients (median = 1.99 pg/mL, n = 15, P = 0.047), and normal donors (median = 1.10 pg/mL, n = 13, p<0.001). No significant difference between normal volunteers and patients with benign pancreatic disease had been seen in the regards of plasma IL-10 levels (P = 0.14).

### Detection of circulating MSLN-specific CD4^+^ and CD8^+^ T cells in cancer and benign patients

**Figure 3:**
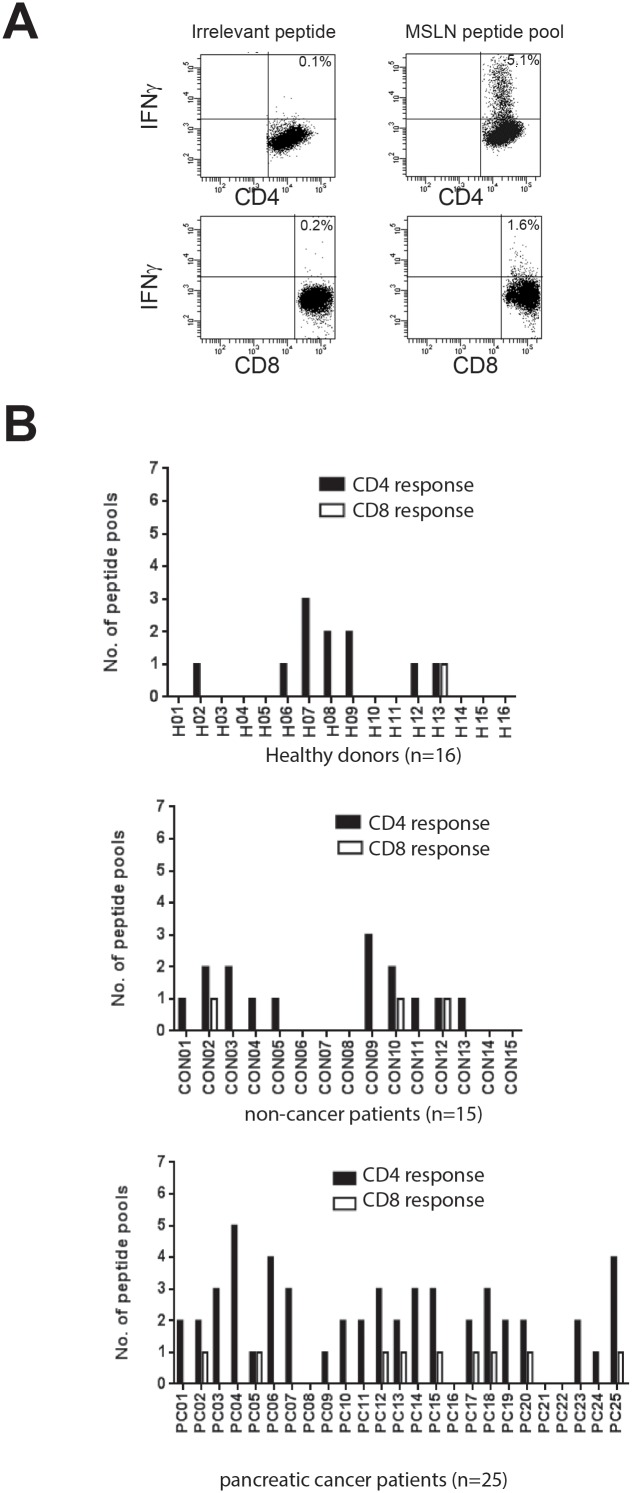
Mesothelin (MSLN)-specific IFN-γ producing CD4^+^ and CD8^+^ T cell response. Representative FACs plots of IFN-γ production by CD4^+^ and CD8^+^ T cells from short term T cell lines following re-stimulation with MSLN-derived peptides or an irrelevant peptide are shown. The production of MSLN-specific IFNγ****by CD4^+^ and CD8^+^ T cells was assessed using intracellular cytokine staining assay. Numbers in the top right quadrants are the percentage of cytokine-producing cells within viable CD4^+^ or CD8^+^ T cells (A). The number of peptide pools (7 peptide pools in total) that stimulated IFN- γ production by CD4^+^ or CD8^+^ T cells isolated from 16 healthy donors, 15 patients with benign pancreatic disease and 25 patients with pancreatic carcinoma are shown (B). An immunological response was defined as a 2-fold increase in the frequency of cytokine-producing cells above control peptides/pools.

Patients’ demographics are described in Table 1 and 2. The clinical stage of the pancreatic cancer was known in 19, and 89% (17 out of 19) had advanced stage disease (stage III/IV). Short-term T cell lines were generated in the presence of MSLN-derived peptide pools (Table 3) and rIL-2 from PBMCs of 25 patients with pancreatic cancer (PC01-PC25), 15 patients with benign pancreatic disease (CON01-CON15), and 16 healthy controls (H01-H20) (Figure 3A and 3B). MSLN specific CD4**^+^** and CD8**^+^** T cells were identified using intracellular cytokine staining assays for IFN-γ and representative dot plots of MSLN-specific CD4**^+^**/CD8**^+^** T cell responses are shown (Figure 3A). An anti-MSLN CD4**^+^** T cell response was observed in 84% of pancreatic cancer patients (21 out of 25), 66.7% of patients with benign disease (10 out of 15) and 43.7% of healthy donors (7 out of 15). The percentage of cancer patients with anti-MSLN T cell response (responders) was significantly higher than that in healthy donors (p = 0.014, χ^2^ test). Anti-MSLN CD8**^+^** T cell responses were detected in 36% of cancer patients (9 out of 25), 20% of benign pancreatic disease patients (3 out of 15) and in only 6.3% of healthy controls (1 out of 16). The number of peptide pools stimulating antigen specific IFN-γ production by CD4**^+^** or CD8**^+^** T cells in 25 patients with pancreatic cancer, 15 patients with benign pancreatic disease and 16 healthy donors are shown (Figure 3B). On average, each cancer patient responded to 2.3 MSLN peptide pools, which was higher than that in patients with benign pancreatic disease (1.2 MSLN peptide pools) and healthy controls (0.7 MSLN peptide pool, p<0.001, one-way ANOVA).

### MSLN-specific CD4^+^ T cell responses are expanded in patients with pancreatic carcinoma

**Figure 4:**
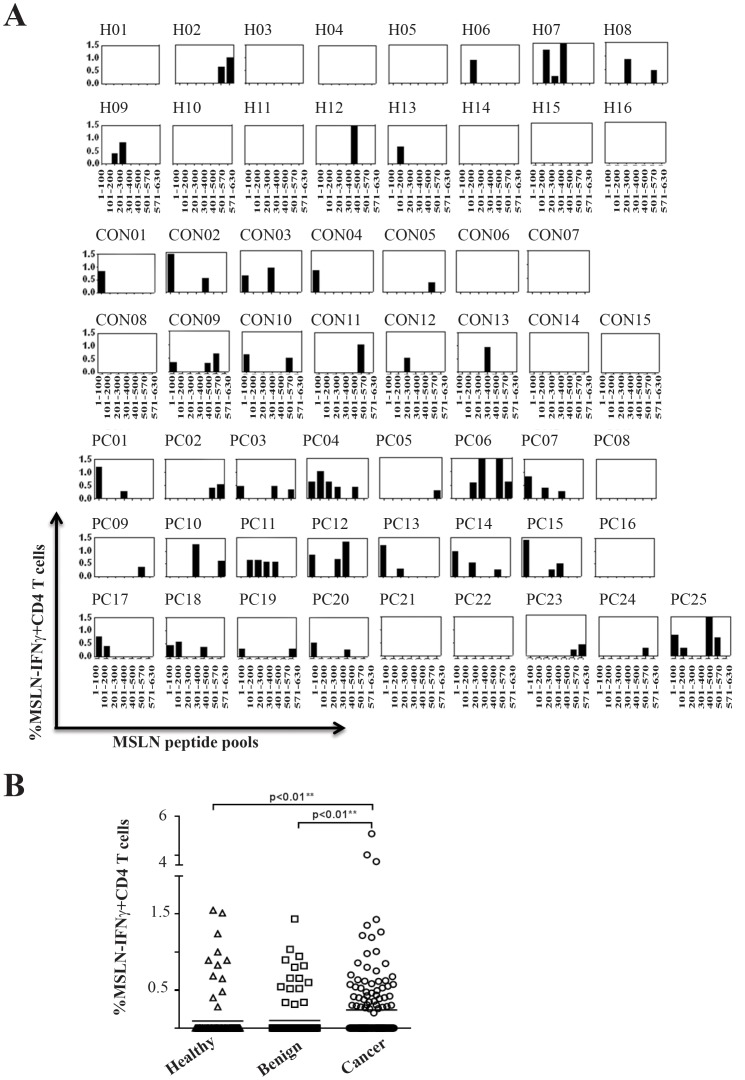
The frequencies of MSLN-specific IFN- γ producing CD4 T cells are shown for healthy donors (H01-H16, n = 16), patients with benign pancreatic disease (CON 01-CON15, n = 15) and patients with pancreatic carcinoma (PC01-PC25, n = 25). T cell lines were stimulated with seven different peptide pools and the frequencies of IFN- γ producing CD4 T cells were analysed using intracellular cytokine staining assays (A). Each symbol represents the percentage of MSLN peptide pool-induced IFN-γ producing CD4^+^ T cells in healthy controls, benign pancreatic disease patients (benign) and patients with pancreatic carcinoma (B). Mann-Whitney U test was used to compare the percentages of CD4 T cell response in the different groups.

The frequency of antigen specific IFN-γ producing CD4**^+^** T cells recognizing MSLN-peptide pools were analysed using an intracellular cytokine staining assay. The percentage of MSLN-specific IFN-γ producing CD4^+^ T cells for healthy donors (H01-H16, n = 16), benign pancreatic disease patients (CON01-CON15, n = 15) and pancreatic cancer patients (P01-PC25, n = 25) are shown in Figure 4A. The results demonstrate that the frequency of circulating anti-MSLN specific CD4^+^ T cells in cancer patients were significantly higher than that observed in the benign pancreatic disease patients (p = 0.0053) and normal controls (p = 0.0004, Figure 4B).

**Figure 5:**
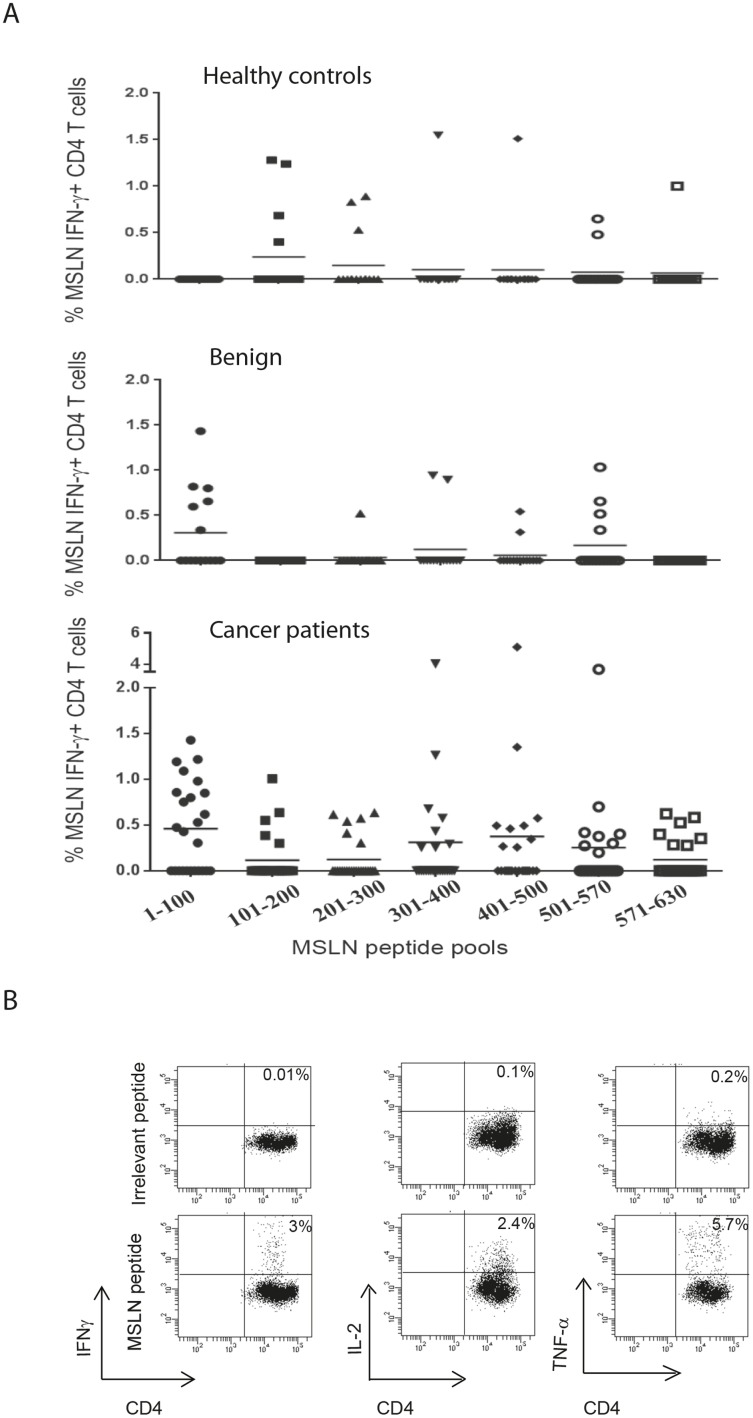
MSLN-specific CD4^+^ T cell responses in patients with pancreatic carcinoma. The frequencies of IFN-**γ** producing CD4^+^ T cells recognizing seven different MSLN-peptide pools are shown in healthy controls, patients with benign pancreatic disease and patients with pancreatic carcinoma, (A). MSLN-specific CD4^+^ T cells recognize MSLN-derived peptides and produced IFN-γ, IL-2 and TNF-α as detected using intracellular cytokine staining assay (B).

CD4**^+^** T cell responses to all 7 different peptide pools were detected in cancer patients. CD4**^+^** T cells recognizing peptide pool 1 was detected in larger number of patients (14 out of 25 patients) than CD4**^+^** T cells recognizing other peptide pools. No CD4**^+^** T cell response recognizing peptide pool 1 was detected in healthy controls (Figure 5A). There was no correlation between the levels of serum MSL and the frequency of MSLN-specific T cell responses (data not shown). In general, the results imply that T cell responses in cancer patients are directed toward a broad repertoire of epitopes within MSLN protein.

### Identification of immunogenic MSLN peptides

To further identify the specific peptides within the peptide pools recognized by the T cells, we assessed IFN-γ production by CD4^+^ and/or CD8^+^ T cells after short term stimulation of T cell lines with individual peptides from various peptide pools. Among the responders, PBMCs from 10 pancreatic cancer (PC01, PC02, PC13, PC17, PC18, PC19, PC22, PC23, PC24, PC25), 6 benign pancreatic disease patients (CON01, CON02, CON04, CON09, CON 10, CON11), and 1 healthy volunteer (H02) were available for further studies. The specific peptide epitope which induced CD4^+^ or CD8^+^ T cell responses are shown in Table 4. It should be noted that very few cells were available for individual peptide analysis from the patients or healthy controls. For example, PBMCs from PC23 were only sufficient to analyse individual peptides within pool 6, but not pool 7. In other cases, we were unable to identify the reacting individual peptides from some patients, including PC02 (reacting to pool 7), because of weak IFN-γ signal with individual peptides. However, cells were available from H02, the only healthy donor with response to pool 7, thus we were able to identified the reacting peptides recognized by PBMCs from this individual (Table 4). CD4 T cells recognized the identified peptide and produced IFN-γ in a dose dependent manner (data not shown). Some peptides were subsequently analyzed for their ability to stimulate intracellular IL-2, TNF-αand TNF-γ****production. The FACS plots shown (Figure 5B) indicate that the CD4^+^ T cells were functional, antigen-specific and able to generate both IL-2 and TNF-α.

**Table 4:** Identified individual reacting peptides.

Peptide pool	Peptide No	sequence	Response type and positive rate	Positive Sample ID
**Pool 1**	Peptide 3	MSLN_21–35_ LLFLLFSLGWVGPSR	CD4 response, positive 4 out of 9 PC patients (44%), and 2 out of 5 (40%) benign patients	PC01, PC13, PC17, PC18, CON 02, CON04
			CD8 response, positive 1 in 9 (11%) PC patients	PC17
	Peptide 10	MSLN_91–105_ QKNVKLSTEQLRCLA	CD4 & CD8, positive 1 in 5 (20%) benign controls; none in PC patients	CON01
**Pool 5**	Peptide 41&42	MSLN_401–415_ VNKGHEMSPQAPRRP MSLN_411–425_ APRRPLPQVATLIDR	CD4 response 1 in 2 (50%) PC patients	PC25
	Peptide 43	MSLN_421–435_ TLIDRFVKGRGQLDK	CD4 response 1 in 2 (50%) PC patients	PC 22
**Pool 6**	Peptide 54	MSLN_531–545_ FMKLRTDAVLPLTVA	CD4 response, positive 2 in 3 (66%) PC patients, negative in 1 benign control and 1 healthy control	PC23, PC24
**Pool7**	Peptide 60&61	MSLN_591–605_ PNGYLVLDLSMQEAL MSLN_601–615_ MQEALSGTPCLLGPG	CD4 response, positive in 1 healthy control, negative in 2 PC patients	H02

### IL-10 blockade enhances MSLN-specific IFN-γ production by CD4^+^ and CD8^+^ T cells

**Figure 6:**
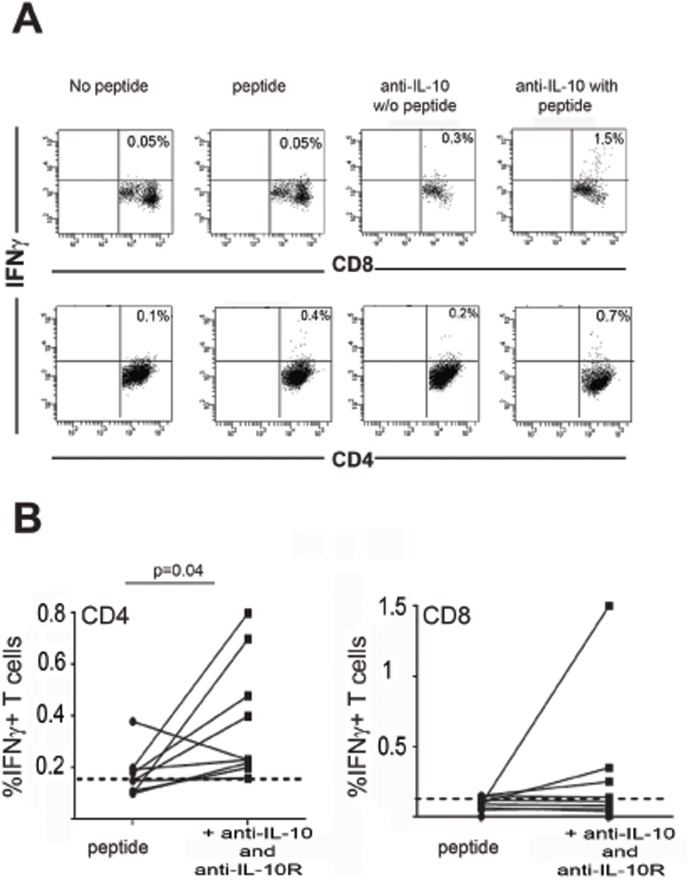
IL-10 blockade unmasked MSLN-specific IFN-γ production by CD4^+^ T cells. Representative FACs plot of peptide specific IFN-γ producing CD8^+^ and CD4^+^ T cells in the presence or absence of anti-IL-10 and anti-IL10Rα mAbs are shown (B). Mann-Whitney U test was used to compare the differences in the percentages of IFN-γ producing cells with or without IL-10 blocking.

IL-10 is an important immunosuppressive cytokine detected in many cancer patients and has the ability to modulate anti-tumour immunity. Since IL-10 levels were significantly elevated in the circulation of pancreatic cancer patients compared to the controls, we tested the influence of IL-10 blockade on the ability of MSLN-specific T cells to produce IFN-γ. PBMCs from 7 patients with pancreatic cancer and 2 patients with benign pancreatic disease were peptide-stimulated in the presence of anti-IL-10 and anti-IL-10 receptor blocking antibodies (added to the cultures on day 1 and 4). T cell lines were re-stimulated with irrelevant or relevant peptide pools prior to intracellular cytokine staining for IFN-γ secretion. Representative dot plots of IFN-γ production by CD4^+^ or CD8^+^T cells following IL-10 blockage are shown in Figure 6A. After peptide stimulation in the presence of anti-IL10 blockade, the frequency of MSLN-specific CD8^+^ T cells was increased in PBMC of 2 out of 7 patients with pancreatic cancer.. Similarly, PBMC from 4 out of 9 patients demonstrated an increased frequency of MLSN-specific IFN-γ producing CD4^+^ T cells after IL-10 blockage (Figure 6B). The results demonstrate that the percentages of IFN-γ producing CD4+ T cells were significantly increased in the presence of the blocking antibodies (p = 0.04). These results suggest that the blockade of IL-10 may restore or promote IFN-γ production in MSLN-specific T cells isolated from patients with pancreatic cancer.

## Discussion

The prognosis for patients with pancreatic cancer remains extremely poor and novel treatment such as T cell based immunotherapy are required to improve survival rate. MSLN is a tumour associated antigen in pancreatic cancer and is a target for immunotherapy. There is little information on the status of naturally occurring MSLN-specific CD4^+^ and CD8^+^ T cell responses in cancer patients. Here, we aimed to assess naturally occurring T cell responses to MSLN in cancer and benign pancreatic disease patients and establish whether there is any possible association between MSLN-T cell responses and the levels of plasma MSLN or cytokines.

MSLN is expressed in more than 90% of pancreatic tumour tissues as demonstrated by immunohistochemistry [26]–[28]. To our knowledge, only one study [31] to date have reported the elevation of circulating MSLN in pancreatic cancer patients compared to healthy controls. Compared to their performance, our study increased the number of healthy and benign pancreatic disease controls, which were considered as risk factors for pancreatic cancer. Meanwhile, majority of the malignant subjects were recruited when the biopsy specimen for diagnosis was taken, which means these patients have not been firmly diagnosed as pancreatic cancer and not received related treatment when we collected the blood samples. Our results confirmed their data and demonstrated that soluble MSLN levels are increased in the plasma of cancer patients and benign pancreatic disease patients compare to that in healthy donors. Similar to their results [31], we could not observe any significant differences in plasma MSLN levels between cancer and benign pancreatic disease patients. However, the mean concentration of MSLN identified in the cancer group was higher than in the benign disease groups.

Elsewhere, elevated serum cytokines and/or chemokines have been observed in cancer patients (such as TNF-α and IL-6), whilst others, like IL-12, are often down-regulated [37]. Here, we focused on the plasma concentration of IL-10, which is regarded as an immune-regulatory cytokines and has the ability to suppress T cell function [38], [39]. The elevation of circulating IL-10 had been previously reported in some cancer patients [40]. Current reports state that circulating IL-10 levels were increased in pancreatic carcinoma compared to healthy controls [41]. To identify whether the levels of IL-10 are increased in pancreatic cancer patients compared to benign pancreatic disease patients, we measured the concentration of this cytokine, known to modulate tumour-specific T cell responses, in the plasma of 34 PC patients, 15 patients with benign pancreatic disease and 13 healthy donors. Consequently, plasma concentrations of IL-10, the important tumour suppressive cytokine, were elevated in PC patients (statistically significant) compared to that of both healthy and benign controls. Thus, our study has further demonstrated that circulating IL-10 levels were significantly increased in pancreatic cancer patients compared to patients with benign pancreatic diseases. Here, we have also shown that mesothelin is increased in benign pancreatic disease patients as compared to healthy donors and IL-10 can inhibit MSLN-specific CD4 T cell responses. These results may suggest that there is possibility of inducing autoimmune pancreatitis by expression of mesothelin in this group of patients. As circulating MSLN was increased in both cancer patients and benign patients, it is possible that pancreatic inflammation and inflammatory mediators can induce MSLN. Further studies are required to evaluate whether inflammatory mediators are able to enhance MSLN expression by pancreatic cells.

We could not establish any association between the levels of elevated plasma IL-10 and the presence or intensity of MSLN-specific T cell responses (data not shown). As we only collected the plasma samples on a single time point, we are not able to describe the change of plasma levels of MSLN and IL-10 during the progression of pancreatic cancer based on our data. However, our data suggested that circulating IL-10 and MSLN levels may undergo quantitative change when malignancy had developed compared to normal status.

Evidence from other studies have shown that detectable T cell responses against MSLN are present in pancreatic cancer patients [31], [42], suggesting that MSLN over-expression may stimulate clinically meaningful self-restricted T cell responses. Most studies to date have focused on CD8^+^ T cell responses. However, there is very little information on the status of CD4^+^ T cell responses, known to be critical for activating and promoting antitumor CD8^+^ T cell memory [18]. We next tried to identify MSLN peptide pool-specific CD8^+^ and CD4^+^ T cell responses and subsequently individual CD4^+^ T cell recognised peptide epitopes using 15-mer amino acid-long overlapping MSLN peptides. The results have confirmed that CD4^+^ and CD8^+^ T cell responses could be expanded in pancreatic cancer patients, and that these patients had an increased frequency of IFN-γ secreting CD4^+^ T cells, compared to the controls. There was no difference in the fluorescent intensity of IFN-γ production between the groups, suggesting that there was no difference in the amounts of cytokine produced, by CD4^+^ or CD8^+^ T cells (data not shown). However, further quantitative analysis of IFN-γ production is required to confirm this observation. In cancer patients, CD4^+^ T cell responses were detected to all seven MSLN-derived peptide pools, suggesting that the response is directed toward a broad repertoire of epitopes within the MSLN protein. Another interesting observation was that no T cell response reacting to pool one was observed in healthy controls, while pool one was the most immunogenic peptide pools in PC patients. Again, we didn’t observe any association between circulating plasma MSLN levels and CD4^+^ T cell responses in pancreatic cancer subjects at the time point analysis. This is in agreement with the results demonstrating that the levels of serum tumour associated antigens, such as Alpha-fetoprotein (AFP) and NY-ESO-1, are not associated with the presence of T cell responses to these antigens [43], [44]. It has been shown that, at least in some malignancies, tumour associated antigens are expressed in tumour tissues but the levels of the tumour associated antigens are not elevated in the serum [45], suggesting that the levels of circulating MSLN do not reflect the amount of MSLN at the tumour site where antigen presenting cells and infiltrating effector T cells can be exposed to MSLN. This may explain why there is no association between serum MSL levels and the presence or absence of MSLN-specific T cell responses. Further studies using the whole MSLN protein as a stimulus are required to stimulate PBMCs and assess T cell responses to the whole antigen. Due to limited number of cells available, we were unable to identify the reacting peptides within the peptide pools in all patients or healthy donors. We had access to further PBMCs from some patients and healthy controls as described in Material and Methods. CD4^+^ and CD8^+^ T cell responses recognizing the individual peptides within the peptide pools were analysed in these patients and controls. The results are presented in Table 4. MSLN_21–35_ LLFLLFSLGWVGPSR was recognized by CD4^+^ T cells in 5 out of 14 patients with pancreatic cancer or benign pancreatic disease. MSLN_401–415_ VNKGHEMSPQAPRRP, MSLN_531–545_ FMKLRTDAVLPLTVA, could also stimulate secretion of IL-2 and TNF-γ. Based on peptide-binding motifs of HLA class II alleles deposited in the SYFPEITHI database, MSLN_21–35_ is predicted to bind to HLA-DRB1*0101, HLA-DRB1*0301, HLA-DRB1*0401, HLA-DRB1*0701, HLA-DRB1*1101 and HLA-DRB1*1501. MSLN_401–415_ is predicted to bind to HLA-DRB1*0101, HLA-DRB1*1101 and HLA-DRB1*1501. MSLN_531–545_ is predicted to bind to HLA-DRB1*0101, HLA-DRB1*0301, HLA-DRB1*0401, HLA-DRB1*0701, HLA-DRB1*1101 and HLA-DRB1*1501.

As we observed significantly increased circulating IL-10 levels in pancreatic cancer patients, compared to the controls, here, we assessed the influence of IL-10 blockade on MSLN-specific T cell responses in 9 pancreatic disease patients. The anti-IL10 blockade (blocking both free IL-10 and IL-10 receptor) increased the frequency of CD8^+^ T cell responses in 2 patients and CD4^+^ T cell responses in 4 patients. This result demonstrates that IL-10 may play a role in modulation of anti-tumour immunity in patients with pancreatic cancer.

In conclusion, we demonstrated that MSLN was immunogenic, reported the dominant region of MSLN required for the induction of specific T cell responses and described an *in vitro* method to promote these T cell responses, which may contribute to the development of MSLN related immunotherapy against pancreatic cancer.
